# Complementary and Inducible *creER*^*T2*^ Mouse Models for Functional Evaluation of Endothelial Cell Subtypes in the Bone Marrow

**DOI:** 10.1007/s12015-024-10703-9

**Published:** 2024-03-04

**Authors:** Michael G. Poulos, Pradeep Ramalingam, Agatha Winiarski, Michael C. Gutkin, Lizabeth Katsnelson, Cody Carter, Laurence Pibouin-Fragner, Anne Eichmann, Jean-Leon Thomas, Lucile Miquerol, Jason M. Butler

**Affiliations:** 1 Department of Medicine, University of Florida Health Cancer Center, Gainesville, FL 32610 USA; 2 Ansary Stem Cell Institute, Division of Regenerative Medicine, Department of Medicine, Weill Cornell Medicine, New York, NY 10065 USA; 3 Université de Paris Cité, Inserm, PARCC, 75015 Paris, France; 4 Department of Molecular and Cellular Physiology, Yale University School of Medicine, New Haven, CT 06510 USA; 5 Cardiovascular Research Center, Department of Internal Medicine, Yale University School of Medicine, New Haven, CT 06511 USA; 6 Department of Neurology, Yale University School of Medicine, New Haven, CT 06511 USA; 7 Paris Brain Institute, Université Pierre et Marie Curie Paris, 06 UMRS1127, Sorbonne Université, Paris Brain Institute, Paris, France; 8 Aix-Marseille Université, CNRS UMR 7288, IBDM, 13288 Marseille, France; 9 Division of Hematology/Oncology, University of Florida, 1333 Center Drive, BH-022D, Gainesville, FL 32610 USA

**Keywords:** Bone Marrow Niche, Arteriole, Sinusoid, Endothelial Cell, Hematopoietic Stem Cell, *Cre* Models

## Abstract

In the adult bone marrow (BM), endothelial cells (ECs) are an integral component of the hematopoietic stem cell (HSC)-supportive niche, which modulates HSC activity by producing secreted and membrane-bound paracrine signals. Within the BM, distinct vascular arteriole, transitional, and sinusoidal EC subtypes display unique paracrine expression profiles and create anatomically-discrete microenvironments. However, the relative contributions of vascular endothelial subtypes in supporting hematopoiesis is unclear. Moreover, constitutive expression and off-target activity of currently available endothelial-specific and endothelial-subtype-specific murine *cre* lines potentially confound data analysis and interpretation. To address this, we describe two tamoxifen-inducible *cre*-expressing lines, *Vegfr3-creER*^*T2*^ and *Cx40-creER*^*T2*^, that efficiently label sinusoidal/transitional and arteriole endothelium respectively in adult marrow, without off-target activity in hematopoietic or perivascular cells. Utilizing an established mouse model in which *cre*-dependent recombination constitutively-activates MAPK signaling within adult endothelium, we identify arteriole ECs as the driver of MAPK-mediated hematopoietic dysfunction. These results define complementary tamoxifen-inducible *creER*^*T2*^-expressing mouse lines that label functionally-discrete and non-overlapping sinusoidal/transitional and arteriole EC populations in the adult BM, providing a robust toolset to investigate the differential contributions of vascular subtypes in maintaining hematopoietic homeostasis.

**Graphical Abstract:**

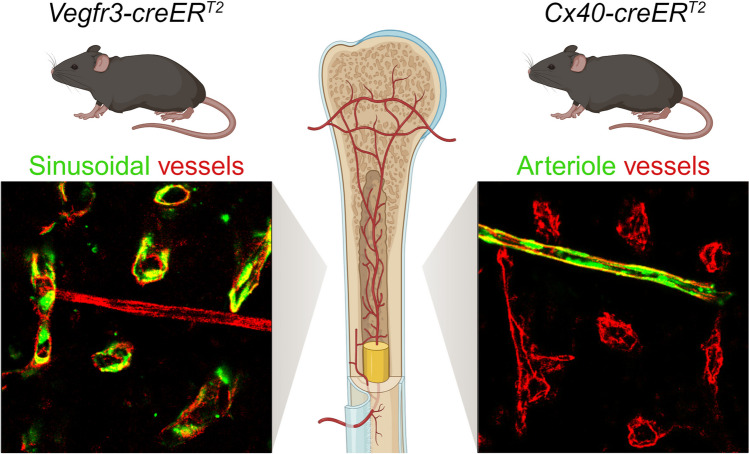

**Supplementary Information:**

The online version contains supplementary material available at 10.1007/s12015-024-10703-9.

## Introduction

Hematopoietic stem cells (HSCs) are multipotent precursors that sit atop a hierarchy of hematopoietic progenitor cells (HPCs) responsible for maintaining balanced blood production throughout life [1, 2]. In adults, hematopoietic stem and progenitor cells (HSPCs) are localized to specialized vascularized niches within the bone marrow (BM) that direct stem cell-fate decisions, including quiescence, self-renewal, and restricted progenitor differentiation [3, 4]. Endothelial cells (ECs) are a critical component of the HSC-supportive BM niche, nucleating perivascular stromal and hematopoietic cells to create an instructive multicellular microenvironment through the production of extrinsic cues that maintain hematopoietic homeostasis and regeneration. Within the BM, the vasculature can be subclassified into high-pressure arterioles, branching into transitional vessels located adjacent to trabecular bone in the metaphysis and near cortical bone, before emptying into a low-pressure sinusoidal capillary network in the central marrow [5]. While arteriole, transitional, and sinusoidal vascular microenvironments are anatomically distinct and can be classified by vessel morphology [6, 7], accompanying perivascular stromal and hematopoietic cell association [4], and endothelial immunophenotypic labeling and gene expression signatures [8, 9], the lack of high-fidelity inducible *cre* systems that allow for targeted genetic manipulations in niche-specific endothelial subtypes have hampered the functional characterization of these vascular subsets.

A diverse array of *cre*-expressing mouse lines have been successfully used to target pan-endothelial and endothelial subtypes [9–14]. However, existing *cre* lines have two limitations: (1) Most pan-endothelial *cre* lines are constitutively expressed and consequently exhibit recombination in HSCs due to their shared developmental ontogeny, and (2) existing *cre* lines targeting vascular subsets exhibit off-target recombination within BM stromal and hematopoietic subsets. These limitations preclude the investigation of the role of vascular-subtype-specific niches in regulating HSPC activity within the adult BM. In this manuscript, we characterize two inducible *cre*-expressing murine lines that faithfully identify adult BM endothelial subpopulations to accurately interrogate the mechanisms of endothelial-HSPC instructive function. Herein, we describe inducible and vascular subtype-specific *Vegfr3-creER*^*T2*^ and *Cx40-creER*^*T2*^ mice that respectively target non-overlapping sinusoidal/transitional and arteriole endothelial populations within the adult BM, with no observable off-target stromal or hematopoietic activity. Using a previously described genetic model of MAPK-activation in the BM vascular niche [15], we demonstrate that *Vegfr3-creER*^*T2*^ and *Cx40-creER*^*T2*^ mice are able to faithfully segregate individual sinusoid/transitional and arteriole MAPK-dependent contributions to hematopoietic dysfunction. Taken together, these model systems provide a platform to discriminate endothelial-borne sinusoidal/transitional and arteriole paracrine signals in the adult BM microenvironment.

## Materials and Methods

### Animals

Murine experiments were performed under the Association for Assessment and Accreditation of Laboratory Animal Care (AAALAC) and National Institutes of Health (NIH) Office of Laboratory Animal Welfare (OLAW) recommendations, in accordance with the University of Florida Institutional Animal Care and Use Committee (IACUC) guidelines. Mice were maintained in specific-pathogen-free housing in NexGen Individually Ventilated Cages (IVC) with HEPA-filtered air exchange (Allentown, Inc.) and fed on PicoLab Rodent Diet 20 (Lab Diet 5053) and water ad libitum. C57BL/6 J-Tg(*Cdh5(PAC)-creER*^*T2*^) [16, 17] and C57BL/6 J-Tg(*Bmx-creER*^*T2*^)1Rha [18] mice were provided by Dr. Ralf Adams at The Max Planck Institute for Molecular Biomedicine. B6.Cg-*Gt(ROSA)26Sor*^*tm6(CAG−ZsGreen1)Hze*^/J (Strain #007906) [19], B6.Cg-*Gt(ROSA)26Sor*^*tm9(CAG−tdTomato)Hze*^/J (Strain #007909) [19], C57BL/6 J-*Gt(ROSA)26Sor*^tm(Map2k1*EGFP)Rsky^/J (Strain #012352) [20], B6.SJL-*Ptprc*^*a*^* Pepc*^*b*^/BoyJ (Strain #002014), and C57BL/6 J mice (Strain #000664) were purchased from The Jackson Laboratory (Bar Harbor, ME). All mice, with the exception of B6.SJL-*Ptprc*^*a*^* Pepc*^*b*^/BoyJ (CD45.1^+^), were maintained on a C57BL/6 J (CD45.2^+^) genetic background.

### *Vegfr3-creER*^*T2*^ Generation

**Fig. 1:**
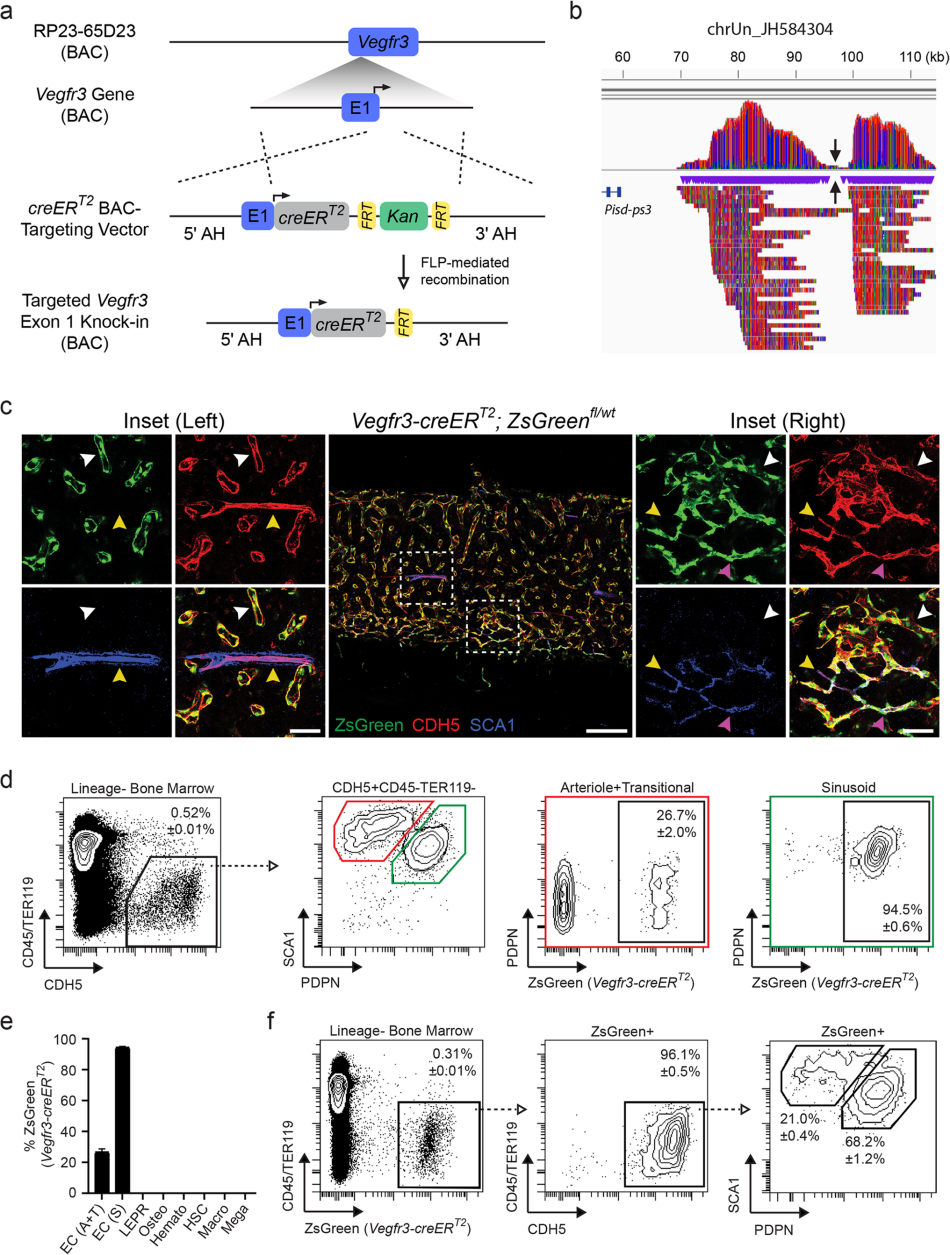
*Vegfr3-creER*^*T2*^ activity in the adult BM is restricted to sinusoidal and transitional endothelium. **a** Schematic of *creER*^*T2*^ BAC-recombineering used to generate *Vegfr3-creER*^*T2*^ transgenic animals. A *creER*^*T2*^ cassette (grey) is knocked-in to exon 1 (E1) of a BAC containing the *Vegfr3* gene (blue) at the transcriptional start site (solid arrow) using adjacent arms of homology (AH). The kanamycin selection cassette (green) was removed via FLP-mediated recombination (open arrow) of flanking FRT sites (yellow) prior to pronuclear injection. **b** Visualization of genomic DNA sequencing reads from *Vegfr3-creER*^*T2*^ transgenic mice mapped to unplaced contig chrUn_JH584304 using Integrative Genome Viewer (IGV). Note: *Vegfr3-creER*^*T2*^ transgene insertion point (inverted black arrows) is approximately 38 kb upstream of the phosphatidylserine decarboxylase - pseudogene 3. **c** Representative images of *Vegfr3-creER*^*T2*^*; ZsGreen*^*fl/wt*^ BM labeled for CDH5 (red), SCA1 (blue), and ZsGreen (green). Insets are denoted by dashed boxes. Arrowheads demarcate vessel type, including sinusoids (white; ZsGreen^+^SCA1^−^CDH5^+^), transitional (magenta; ZsGreen^+^SCA1^+^CDH5^+^), and arterioles (yellow; ZsGreen^−^SCA1^+^CDH5^+^). Scale bars for the central image (200 μm) and insets (50 μm) are noted. **d** Representative flow plots to quantify *Vegfr3-creER*^*T2*^ activity in BM EC subtypes; arteriole and transitional (red box) and sinusoids (green box) are indicated. Open arrow/dashed line illustrates gating progression. Percentages represent AVE ± SEM; *N* = 4. **e** Quantification of *Vegfr3-creER*^*T2*^*; ZsGreen*^*fl/wt*^ activity in BM cellular subsets, including arteriole and transitional (EC; A + T) and sinusoidal (EC; S) endothelium, LEPR^+^ mesenchymal cells, osteoblasts (Osteo), pan-hematopoietic cells (Hemato), hematopoietic stem cells (HSCs), macrophages (Macro), and megakaryocytes (Mega). Percentages represent AVE ± SEM; *N* = 4. **f** Representative flow plots assessing endothelial populations in ZsGreen^+^ cells derived from *Vegfr3-creER*^*T2*^*; ZsGreen*.^*fl/wt*^ BM. Open arrow/dashed line illustrates gating progression. Percentages represent AVE ± SEM; *N* = 4

*Vegfr3-creER*^*T2*^ transgenic animals were generated following a previously published strategy [21]. The *Vegfr3-creER*^*T2*^ bacterial artificial chromosome (BAC)-targeting vector was provided by Jean-Leon Thomas; in short, the described *Vegfr3* BAC-targeting vector containing a *Venus* (YFP) fluorescent cassette [21] was replaced with a tamoxifen-inducible *creER*^*T2*^ cassette in-frame with the *Vegfr3* start codon in exon 1. Recombineering bacterial strains (SW102 and SW105) were obtained from the National Cancer Institute at Frederick [22]. The ~ 240 kilobase (kb) murine C57BL/6 *Vegfr3*-containing BAC (RP23-65D23) was obtained from CHORI (https://bacpacresources.org). Recombineering protocols were followed as detailed [23] and briefly described below. To target the *Vegfr3*-containing BAC, the *creER*^*T2*^ BAC-targeting vector was digested with PacI/AscI (New England Biolabs) and the resulting 4497 bp fragment was purified from a 0.9% TAE/agarose gel using the QIAquick Gel Extraction Kit (Qiagen) according to the manufacturer’s recommendations. The linearized *creER*^*T2*^ BAC-targeting vector was transformed into RP23-65D23-containing SW102 cells and selected for Kanamycin resistance to identify recombinants (Fig. 1a). Successfully-targeted BACs were purified using the Nucleobond BAC 100 Kit (Takara), stably transformed into SW105 cells by selecting for BAC-specific chloramphenicol resistance (i.e. pBACE3.6 backbone), and the Kanamycin resistance cassette was excised via arabinose-induced FLP-mediated recombination (Fig. 1a). Using replica plating, chloramphenicol^+^ kanamycin^−^ clones were selected, purified, and transformed into SW102 cells. An ampicillin targeting cassette was amplified from pBluescript using primers with AscI restriction sites and arms of homology flanking the single *loxP* site located in the pBACE3.6 vector. The ampicillin PCR product was transformed into chloramphenicol^+^ kanamycin^−^ SW102 cells and successful recombinants with the removed *loxP* were selected using ampicillin. The finalized *creER*^*T2*^-targeted RP23-65D23 construct was linearized with AscI and fractionated using a Sepharose CL-4B (Sigma-Aldrich) chromatography column (equilibrated to 100 mM NaCl, 10 mM Tris–HCl (pH 7.5), and 0.25 mM EDTA); fractions containing intact targeted-BAC construct at ~ 245 kb were confirmed using pulse-field gel electrophoresis. Transgenic animals were generated at the University of Michigan – Transgenic Animal Core (https://medresearch.umich.edu/office-research/about-office-research/biomedical-research-core-facilities/transgenic-animal-model) via pronuclear injection of linearized *creER*^*T2*^-targeted RP23-65D23 into fertilized C57BL/6 J oocytes. Founders were screened by PCR using Hot Start *Taq* DNA polymerase (New England BioLabs) with *cre*-specific primers 5ʹ-atgtccaatttactgaccgtacacca-3ʹ and 5ʹ-acgatgaagcatgtttagctggccca-3ʹ (Integrated DNA Technologies) according to the manufacturer’s recommendations.

### *Vegfr3-creER*^*T2*^ Transgene Mapping

The *Vegfr3-creER*^*T2*^ BAC genomic insertion site was determined by long-read sequencing of high molecular weight (HMW) leukocyte DNA. In short, HMW DNA was purified from 1 mL of red blood cell (RBC)-lysed peripheral blood from a male *Vegfr3-creER*^*T2*^ heterozygous animal using the Monarch HMW DNA Extraction Kit for Cells & Blood (New England BioLabs) according to the manufacturer’s recommendation. The resulting HMW genomic DNA was sheared to a size of ~ 20 kb using a g-TUBE (Covaris) according to the manufacturer’s suggestions. The sequencing library was prepared using the Ligation Sequencing Kit V14 (Oxford Nanopore Technologies). Briefly, 1 μg of DNA was repaired and end-prepped using the NEBNext Ultra II End Repair/dA-tailing Module (New England Biolabs). Sequencing adapters were ligated to the DNA ends and the adapted library was cleaned to remove fragments shorter than 3 kb. Twenty-two fmol of library DNA was loaded onto an R10.4.1 flow cell (FLO-PRO114M) and sequencing was carried out on a PromethION (Oxford Nanopore Technologies) instrument. Base calling was carried out directly on the device with the MinKNOW software using the high-accuracy setting and a minimum quality score of 8. Long DNA sequencing reads (5.9 M reads, N50 read length = 12,705 bp, average genome coverage = 20X) were split into non-overlapping contiguous 500 bp fragments and independently mapped to the mouse genome (C57BL/6 J; reference version GRCm39) and transgene sequence using minimap2 [24]. The identified 923 long reads containing at least two 500 bp fragments mapping to both the mouse genome and the transgene were mapped back to the mouse genome and custom scripts were used to identify the region with the highest number of aligned reads. The integration site was identified on unplaced contig chrUn_JH584304, representing two contiguous peaks of high coverage. BAM files produced by the alignment of the fragments to the mouse genome were visualized using Integrative Genome Viewer (IGV) [25] to verify the location of the integration site and to generate Fig. 1b.

### *Cx40-creER*^*T2*^ Backcross

**Fig. 2:**
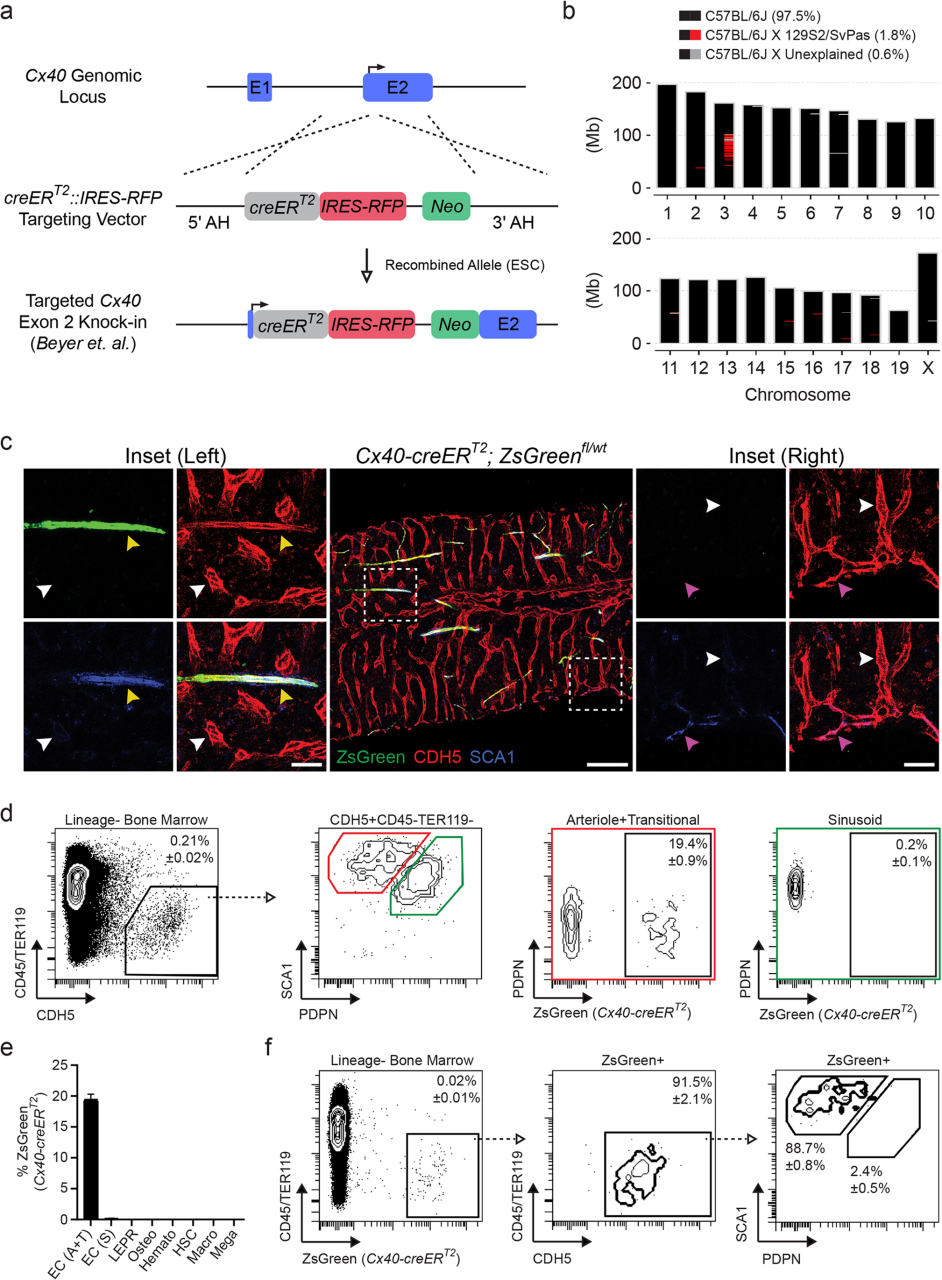
*Cx40-creER*^*T2*^ activity in the adult BM is restricted to arteriole endothelium. **a** Overview of bicistronic *creER*^*T2*^ (grey)::*IRES-RFP* (red) and *PGK*-driven *Neo* (green) embryonic stem cell (ESC) targeting of the endogenous *Cx40* gene (blue) at the exon 2 (E2) transcriptional start site (solid arrow) using adjacent arms of homology (AH); schematic adopted from *Beyer *et al. **b** Chromosomal Ideogram of *Cx40-creER*^*T2*^ mice following backcrossing to a C57BL/6 J genetic background. Recipient C57BL/6 J congenic (black), ESC-targeted *Cx40* allele located on chromosome 3 (red; 129S2/SvPas origin), and unassigned regions (grey) are detailed. Megabase (Mb). **c** Representative images of *Cx40-creER*^*T2*^*; ZsGreen*^*fl/wt*^ BM labeled for CDH5 (red), SCA1 (blue), and ZsGreen (green). Insets are denoted by dashed boxes. Arrowheads demarcate vessel type, including sinusoids (white; ZsGreen^−^SCA1^−^CDH5^+^), transitional (magenta; ZsGreen^−^SCA1^+^CDH5^+^), and arterioles (yellow; ZsGreen^+^SCA1^+^CDH5^+^). Scale bars for the central image (200 μm) and insets (50 μm) are noted. **d** Representative flow plots to quantify *Cx40-creER*^*T2*^ activity in BM EC subtypes; arteriole and transitional (red box) and sinusoids (green box) are indicated. Open arrow/dashed line illustrates gating progression. Percentages represent AVE ± SEM; *N* = 4. **e** Quantification of *Cx40-creER*^*T2*^*; ZsGreen*^*fl/wt*^ activity in BM cellular subsets, including arteriole and transitional (EC; A + T) and sinusoidal (EC; S) endothelium, LEPR^+^ mesenchymal cells, osteoblasts (Osteo), pan-hematopoietic cells (Hemato), hematopoietic stem cells (HSCs), macrophages (Macro), and megakaryocytes (Mega). Percentages represent AVE ± SEM; *N* = 4. **f** Representative flow plots assessing endothelial populations in ZsGreen^+^ cells derived from *Cx40-creER*^*T2*^*; ZsGreen*.^*fl/wt*^ BM. Open arrow/dashed line illustrates gating progression. Percentages represent AVE ± SEM; *N* = 4

Outbred *Gja5*^*tm2(cre/ERT2,RFP)Lumi*^ (*Cx40-creER*^*T2*^) knock-in mice were generated by and obtained from Dr. Lucile Miquerol at The Developmental Biology Institute of Marseilles [26] and backcrossed to C57BL/6 J for six successive generations using speed congenics. In short, purified genomic DNA from resulting pups were screened between generations using the miniMUGA SNP Array (Neogen) to identify animals with the highest recipient genomic percentage. Selected animals were bred back to C57BL/6 J animals. The congenic C57BL/6 J background in the N6 generation was confirmed (Fig. 2b) prior to generating B6.Cg-*Gt(ROSA)26Sor*^*tm6(CAG−ZsGreen1)Hze*^/J or C57BL/6 J-*Gt(ROSA)26Sor*^tm(Map2k1*EGFP)Rsky^/J models.

### Tamoxifen Induction

To induce *creER*^*T2*^-mediated recombination in heterozygous B6.Cg-*Gt(ROSA)26Sor*^*tm6(CAG−ZsGreen1)Hze*^ /J (*ZsGreen*^*fl/wt*^) or B6.Cg-*Gt(ROSA)26Sor*^*tm9(CAG−tdTomato)Hze*^/J (*tdTomato*^*fl/wt*^) reporter animals, adult (8 weeks) *Vegfr3-creER*^*T2*+^*; ZsGreen*^*fl/wt*^, *Cx40-creER*^*T2*+^; *ZsGreen*^*fl/wt*^, and *Bmx-creER*^*T2*^; *tdTomato*^*fl/wt*^ mice were fed Custom Teklad PicoLab Rodent Diet 20 (Lab Diet 5053) supplemented with 5% w/w Sucrose and 0.25% Tamoxifen ad libitum for four weeks. Mice were allowed to recover for four weeks prior to analysis. Age- and sex-matched littermate controls (*creER*^*T2−*^; *ZsGreen*^*fl/wt*^ or *creER*^*T2−*^; *tdTomato*^*fl/wt*^) that underwent the same Tamoxifen regimen were used as experimental controls.

To induce *creER*^*T2*^-mediated recombination in homozygous C57BL/6 J-*Gt(ROSA)26Sor*^tm(Map2k1*EGFP)Rsky^/J (*Mapk*^*fl/fl*^) animals, adult (8 weeks) *Vegfr3-creER*^*T2*+^*; Mapk*^*fl/fl*^ (R3-MAPK), *Cx40-creER*^*T2*+^*; Mapk*^*fl/fl*^ (Cx40-MAPK), and *Cdh5-creER*^*T2*+^*; Mapk*^*fl/fl*^ (Cdh5-MAPK) mice were fed Custom Teklad PicoLab Rodent Diet 20 (Lab Diet 5053) supplemented with 5% w/w Sucrose and 0.25% Tamoxifen ad libitum for four weeks. Mice were allowed to recover for four weeks prior to analysis. Age- and sex-matched littermate controls (*creER*^*T2−*^; *Mapk*^*fl/fl*^) that underwent the same Tamoxifen regimen were used as experimental controls.

### Microscopy

Endothelium were labeled in situ in tamoxifen-induced adult *Vegfr3-creER*^*T2*+^, *Cx40-creER*^*T2*+^, and *Bmx-creER*^*T2*+^ reporter animals (*ZsGreen*^*fl/wt*^ or *tdTomato*^*fl/wt*^) by retro-orbital sinus injections with an αCDH5 antibody (Supplementary Table 1). Mice were euthanized 10 min post-injection and femurs, liver, and spleen samples were collected and fixed overnight with 4% paraformaldehyde (in PBS; pH 7.2) at 4 °C. Femurs were washed three times (15 min/wash) with PBS (pH 7.2) at room temperature, decalcified in 10% EDTA in PBS (pH 7.2) for 72 h at room temperature, and normalized to 30% sucrose (in PBS; pH 7.2) for 72 h at 4 °C. Liver and spleen samples were washed three times (15 min/wash) with PBS (pH 7.2) at room temperature and normalized to 30% sucrose (in PBS; pH 7.2) for 72 h at 4 °C. Tissues were then embedded in 1:1 mixture of Tissue-Tek O.C.T. (Sakura) and 30% sucrose (in PBS; pH 7.2) and snap frozen in N_2_(l). To expose the marrow cavity for whole mount analysis, femurs were shaved longitudinally using a cryostat (Leica 3050S) and washed three times (5 min/wash) in PBS (pH 7.2) to remove excess O.C.T. Exposed marrow was then permeabilized in PBS (pH 7.2) with 20% (v/v) Normal Goat Serum (Jackson ImmunoResearch) and 0.5% (v/v) Triton X-100 (Sigma-Aldrich) for 2 h at room temperature and stained with an αSCA1 antibody for 48 h at 4 °C (Supplementary Table 1). For liver and spleen, sections were cut (12 μm) using a cryostat (Leica 3050S) and washed three times (5 min/wash) in PBS (pH 7.2) to remove excess O.C.T., and permeabilized in PBS (pH 7.2) with 20% (v/v) Normal Goat Serum (Jackson ImmunoResearch) and 0.5% (v/v) Triton X-100 (Sigma-Aldrich) for 30 min at room temperature. Tissues were washed three times (15 min/wash) in PBS (pH 7.2) and stained with DAPI (Biolegend) at 1 μg/mL in PBS (pH 7.2) for 15 min at room temperature (where applicable). Tissues were mounted with ProLong Gold (ThermoFisher Scientific) and imaged using a Nikon C2 confocal LASER-scanning microscope; 40 μm Z-stack images were acquired and denoised (Denoise.ai) and rendered into a maximum intensity projection using NIS Elements software (Nikon).

### Whole Bone Marrow Isolation

Individual femurs were disassociated using a mortar and pestle in PBS (pH 7.2) + 0.5% BSA (w/v) + 2 mM EDTA and filtered (40 μm; Corning) to ensure a single cell suspension. Cell counts were determined using a Hemocytometer (Reichert Bright-Line; Hausser) and Trypan Blue (ThermoFisher Scientific) according to the manufacturer’s recommendations. For HSPC analysis by flow cytometry, whole bone marrow (WBM) suspensions were depleted of terminally-differentiated hematopoietic cells using the murine-specific Lineage Cell-Depletion Kit (MiltenyiBiotec) according to the manufacturer’s recommendations.

### Bone Marrow Digestion

Individual femurs were disrupted using a mortar and pestle in Hanks Balanced Salt Solution (Corning) + 10 mM HEPES (pH 7.2) and enzymatically disassociated under gentle agitation with 2.5 mg/mL Collagenase A (Roche) and 1 Unit/mL Dispase II (Roche) for 20 min at 37 °C. Resulting cell suspensions were filtered (40 μm; Corning) and washed using ten times digestion volume with PBS (pH 7.2) + 0.5% BSA (w/v) + 2 mM EDTA. Cell counts were determined using a Hemocytometer (Reichert Bright-Line; Hausser) and Trypan Blue (ThermoFisher Scientific) according to the manufacturer’s recommendations. To enrich for EC, stromal cell, and HSPC fractions, digested BM was depleted of terminally-differentiated hematopoietic cells using the murine-specific Lineage Cell-Depletion Kit (MiltenyiBiotec) according to the manufacturer’s recommendations.

### Liver/Spleen Digestion

Individual liver or spleen samples were minced to ~ 1mm^3^ using sterile scalpels and enzymatically disassociated under gentle agitation in Hanks Balanced Salt Solution (Corning) + 10 mM HEPES (pH 7.2) with 2.5 mg/mL Collagenase A (Roche) and 1 Unit/mL Dispase II (Roche) for 30 min at 37 °C. Resulting cell suspensions were filtered (40 μm; Corning) and washed using ten times digestion volume with PBS (pH 7.2) + 0.5% BSA (w/v) + 2 mM EDTA. Cell counts were determined using a Hemocytometer (Reichert Bright-Line; Hausser) and Trypan Blue (ThermoFisher Scientific) according to the manufacturer’s recommendations.

### Flow Cytometry

For all flow cytometry, 2 × 10^6^ total cells were blocked with αCD16/CD32 (Supplementary Table 1) in 200 μL PBS (pH 7.2) + 0.5% BSA (w/v) + 2 mM EDTA for 10 min at 4 °C and stained with the appropriate antibodies for 45 min at 4 °C. Stained cells were washed with 1 mL PBS (pH 7.2) + 0.5% BSA (w/v) + 2 mM EDTA and fixed in in PBS (pH 7.2) + 2 mM EDTA with 1% paraformaldehyde. All samples were analyzed on a BD LSRFortessa SORP using BD FACSDiva Software (v9.0).

### Recombination Quantification

To quantify *creER*^*T2*^-mediated recombination in tamoxifen-induced adult *Vegfr3-creER*^*T2*+^, *Cx40-creER*^*T2*+^, and *Bmx-creER*^*T2*+^ reporter animals (*ZsGreen*^*fl/wt*^ or *tdTomato*^*fl/wt*^), WBM (i.e. hematopoietic cells), digested WBM (i.e. LEPR^+^ cells/osteoblasts), digested/lineage-depleted BM cells (i.e. endothelium/HSPCs), and digested liver/spleen cells were stained with antibodies described in Supplementary Table 1 and analyzed by flow cytometry.

### HSPC Analysis

HSPC populations were quantified by flow cytometry from WBM stained with antibodies described in Supplementary Table 1.

### Colony Forming Assays

Based on Hemocytometer counts, 7.5 × 10^4^ total WBM cells in 300 μL Low-Glucose DMEM (ThermoFisher Scientific) were added to 3 mL MethoCult GF M3434 (StemCell Technologies) and plated in duplicate (2.5 × 10^4^ cells/well) on low-adherent 6-well plates. Cells were incubated at 37 °C 5% CO_2_ and scored for hematopoietic progenitor colony-forming units ten days post-plating using an SZX16 Stereo Microscope (Olympus) according to the manufacturer’s guidelines.

### Peripheral Blood

To examine multilineage donor engraftment or complete blood counts, mice were bled via the retro-orbital sinus using 75 mm heparinized capillary tubes (Kimble-Chase) into microfuge tubes with PBS (pH 7.2) + 10 mM EDTA and analyzed as described.

### Complete Blood Counts

Complete blood counts were quantified using an Element HT5 (Heska) veterinary hematological analyzer according to the manufacturer’s recommendations.

### Competitive Transplantation

For competitive transplantations, 5 × 10^5^ CD45.2^+^ donor BM cells + 5 × 10^5^ CD45.1^+^ competitor BM cells isolated from 16-week-old mice were intravenously injected into adult (12 weeks) CD45.1^+^ recipient mice pre-conditioned with split-dose total body irradiation (2 × 475 cGy; RadSource RS2000 Small Animal X-Ray Irradiator). For long-term (16 weeks post-transplantation) multilineage engraftment quantification by flow cytometry, peripheral blood was depleted of red blood cells using RBC lysis buffer (Biolegend) according to the manufacturer’s recommendations, stained with antibodies described in Supplementary Table 1, and analyzed by flow cytometry.

### Hematopoietic Recovery

For myelosuppression, control (*creER*^*T2−*^*; Mapk*^*fl/fl*^) and experimental (*creER*^*T2*+^*; Mapk*^*fl/fl*^) mice were subjected to single-dose total body irradiation (450 cGy; RadSource RS2000 Small Animal X-Ray Irradiator) and bled weekly to determine complete blood count recovery kinetics. Non-irradiated baseline counts were determined two weeks prior to myelosuppression.

### Statistical Analysis

Experimental significance was determined using Prism 9.5.1 Software (Graphpad). Statistical analysis and parameters are indicated in individual figure legends.

## Results

### *Vegfr3-creER*^*T2*^ Targets Sinusoidal and Transitional Endothelium in the Bone Marrow

To generate an inducible sinusoid-specific *cre*-expressing mouse model, we utilized a C57BL/6 J-derived bacterial artificial chromosome (BAC) containing the *Vegfr3* gene with approximately 130 kb upstream and 60 kb downstream genomic DNA sequence. This BAC was previously used to generate *Vegfr3-Yfp* reporter mice [21] that discriminate sinusoidal endothelium from arterioles in adult BM [27]. Using bacterial recombineering, a *creER*^*T2*^ cassette was introduced in-frame downstream of the *Vegfr3* exon 1 start codon (Fig. 1a). The residual Kanamycin selection cassette was removed via FLP-mediated recombination prior to targeted-BAC linearization and pronuclear injection into fertilized C57BL/6 J zygotes. Resulting *Vegfr3-creER*^*T2*+^ offspring were maintained on a C57BL/6 J background. The transgene insertion site was mapped to unplaced scaffold chromosome chrUN_JH584304 approximately 40 kb upstream of phosphatidylserine decarboxylase - pseudogene 3 (*Pisd-ps3*) (Fig. 1b), avoiding the disruption of any known protein-coding genes. We next sought to evaluate the fidelity of *Vegfr3-creER*^*T2*+^ mice to mark sinusoidal endothelium in the adult BM microenvironment.

To evaluate *Vegfr3-creER*^*T2*^ activity in the BM, adult *Vegfr3-creER*^*T2*+^; *ZsGreen*^*fl/wt*^ reporter mice were induced with tamoxifen-supplemented feed and assessed for *ZsGreen* expression within the BM by immunofluorescence (IF) imaging and flow cytometry. To discriminate patent vasculature, pan-endothelium were labeled in vivo by intravital αCDH5 staining that excludes lymphatic vessels [28]. In combination with αCDH5 staining, high expression levels of *Sca1* on arteriole endothelium make it an effective marker to delineate arteriole from sinusoidal vasculature in the BM [29, 30]. IF analysis revealed that *Vegfr3-creER*^*T2*^ mice labeled ZsGreen^+^CDH5^+^SCA1^−^ sinusoidal endothelium (Fig. 1c; left inset-white arrowhead) that were distinct from ZsGreen^−^CDH5^+^SCA1^+^ arteriole endothelium (Fig. 1c; left inset-yellow arrowhead). In addition to sinusoids, *Vegfr3-creER*^*T2*^ mice marked ZsGreen^+^CDH5^+^SCA1^+^ transitional endothelium (Fig. 1c; right inset-magenta arrowhead). Transitional endothelium are distinct from ZsGreen^−^CDH5^+^SCA1^+^ arterioles (Fig. 1c; right inset-yellow arrowhead) and ZsGreen^+^CDH5^+^SCA1^−^ sinusoids (Fig. 1c; right inset-white arrowhead). We confirmed *Vegfr3-creER*^*T2*^ specificity in BM cellular niche constituents and hematopoietic cell populations by flow cytometry. Using PDPN and SCA1 to delineate EC subtypes in enzymatically-dissociated WBM [9], *Vegfr3-creER*^*T2*^ efficiently labeled 94.5% of immunophenotypic PDPN^+^SCA1^dim^ sinusoidal endothelium within the pan-endothelial CDH5^+^CD45^−^TER119^−^ population (Fig. 1d, e). Interestingly, *Vegfr3-creER*^*T2*^ activity was also observed in 26.7% of the PDPN^−/dim^SCA1^bright^ ECs (Fig. 1d, e), revealing that the previously described PDPN^−/dim^SCA1^bright^ “arterioles” described by flow cytometry contain both arterioles and transitional endothelium identified by imaging (Fig. 1c). Gating on total ZsGreen^+^CD45^−^TER119^−^ WBM cells confirmed *Vegfr3-creER*^*T2*^ vascular endothelial specificity, in which 96.1% of ZsGreen^+^ cells were CD45^−^TER119^−^CDH5^+^ (Fig. 1f). Additionally, ZsGreen^+^CDH5^+^ mark transitional endothelium as a subset within PDPN^−/dim^SCA1^bright^ endothelial population (Fig. 1d, f). *Vegfr3-creER*^*T2*^ activity is notably absent in immunophenotypic LEPR^+^ mesenchymal stem cells (MSCs), CD51^+^SCA1^−^ osteoblasts, CD45^+^ pan-hematopoietic cells, lineage^−^cKIT^+^SCA1^+^CD48^−^CD150^+^ HSCs, GR1^Low/−^F4/80^+^CD115^−^ macrophages, and CD41^+^ megakaryocytes (Fig. 1e and Supplementary Fig. 1a-f). In addition to the BM, *Vegfr3-creER*^*T2*^ activity is also observed in CDH5^+^ liver and spleen sinusoidal endothelium (Supplementary Fig. 2a and Supplementary Fig. 3a). Moreover, *Vegfr3-creER*^*T2*^ activity is restricted to the vasculature and not observed in either hematopoietic or stromal compartments (Supplementary Fig. 2b and Supplementary Fig. 3b).

### *Bmx-creER*^*T2*^ Labels Arteriole Endothelium and LEPR^+^ Cells in the Bone Marrow

We next evaluated *Bmx-creER*^*T2*^ transgenic animals [18], an established inducible *creER*^*T2*^ model system used to label BM arterioles [9, 31–33]. To confirm their specificity, we examined BM from tamoxifen-induced adult *Bmx-creER*^*T2*+^*; tdTomato*^*fl/wt*^ reporter mice. *Bmx-creER*^*T2*^ mice efficiently labeled BM arteriole endothelium (Supplementary Fig. 4a; right inset-white arrowhead), but also marked CDH5^−^ non-endothelial perisinusoidal cells (Supplementary Fig. 4a; right inset-yellow arrowhead) and interstitial cells (Supplementary Fig. 4a; left inset-magenta arrowhead). While flow cytometry confirmed that *Bmx-creER*^*T2*^ labeled a subset of vascular endothelium in the BM (Supplementary Fig. 4b), the vast majority of tdTomato^+^ cells (~ 80%) were non-endothelial (Supplementary Fig. 4c). Because the perisinusoidal and interstitial tdTomato^+^CDH5^−^ staining pattern is reminiscent of previously described *Lepr-cre* activity in the BM [34–36], we examined *Bmx-creER*^*T2*^ activity in LEPR^+^ cells by flow cytometry. While *Bmx-creER*^*T2*^ activity was observed in only 7.1% of LEPR^+^CD45^−^TER119^−^CDH5^−^ MSCs (Supplementary Fig. 4d), the vast majority of tdTomato^+^CD45^−^TER119^−^CDH5^−^ cells (i.e. non-endothelial and non-hematopoietic) were LEPR^+^ (Supplementary Fig. 4e). Alongside vascular endothelium in the BM microenvironment, LEPR^+^ MSCs cooperatively support HSPC function and niche integrity through the expression of hematopoietic-supportive factors [34, 36–44]. While *Bmx-creER*^*T2*^ efficiently labels BM arterioles, the potential for off-target effects in LEPR^+^ cells should be taken into consideration during experimental design and data interpretation.

### *Cx40-creER*^*T2*^ Activity is Restricted to Arteriole Endothelium in the Bone Marrow

To identify an arteriole-specific expression pattern within the BM, we analyzed previously published RNA sequencing data of sort-purified BM arteriole and sinusoidal ECs by *Xu et. al.*, revealing *Gja5* (*Connexin40/Cx40*) as a candidate gene [9]. Cx40 is a member of the connexin family of transmembrane gap junction proteins that mediate intercellular crosstalk and coordinate multicellular functionality through ion exchange [45–47]. In the murine vasculature, *Cx40* is expressed in large artery and arteriolar endothelium [48, 49]. To establish a BM arteriole-specific model system, we utilized a mouse line previously published by *Beyer et. al.* in which a *creER*^*T2*^*::IRES-RFP* was knocked-in the endogenous *Cx40* gene in-frame with the canonical translational start site located in exon 2 (Fig. 2a) [26]. Relatively weak RFP signal in *Cx40-creER*^*T2*^*::IRES-RFP* mice does not interfere with immunohistochemistry or flow cytometry readouts in the red channel and requires an RFP-specific antibody for detection (data not shown) [26]. Outbred *Cx40-creER*^*T2*^ mice were backcrossed to C57BL/6 J recipients using a speed congenics approach for six generations; mating pair selection and backcrosses were confirmed using the miniMUGA array-based platform (Fig. 2b) [50]. To evaluate *Cx40-creER*^*T2*^ activity in the BM, adult *Cx40-creER*^*T2*+^*; ZsGreen*^*fl/wt*^ reporter mice were induced with tamoxifen-supplemented feed and assessed for *ZsGreen* expression by imaging and flow cytometry. BM IF analysis demonstrated that *Cx40-creER*^*T2*^ specifically mark ZsGreen^+^CDH5^+^SCA1^+^ arteriole endothelium (Fig. 2c; left inset-yellow arrowhead), with no observable activity in the sinusoidal compartment (Fig. 2c; left inset-white arrowhead). Notably, *Cx40-creER*^*T2*^ activity was not observed in transitional endothelium (Fig. 2c; right inset-magenta arrowhead), making *Vegfr3-creER*^*T2*^ and *Cx40-creER*^*T2*^ mice compatible systems to label discrete endothelial subtypes in the adult BM. Furthermore, quantification of *Cx40-creER*^*T2*^ activity of enzymatically-dissociated WBM using flow cytometry revealed ~ 20% ZsGreen^+^ arteriole staining within the PDPN^−/dim^SCA1^bright^ gate containing both arteriole and transitional ECs, while no signal was detected in the PDPN^+^SCA1^dim^ sinusoidal population (Fig. 2d, e). Among total ZsGreen^+^CD45^−^TER119^−^ BM cells, ZsGreen^+^ cells primarily fell within the CDH5^+^ pan-endothelial population and subsequently the PDPN^−/dim^SCA1^bright^ gate arteriole/transitional endothelial gate (Fig. 2f). *Cx40-creER*^*T2*^ activity was not detected in immunophenotypically-defined LEPR^+^ MSCs, CD51^+^SCA1^−^ osteoblasts, CD45^+^ pan-hematopoietic cells, lineage^−^cKIT^+^SCA1^+^CD48^−^CD150^+^ HSCs, GR1^Low/−^F4/80^+^CD115^−^ macrophages, or CD41^+^ megakaryocytes (Fig. 2e and Supplementary Fig. 5a-f). *Cx40-creER*^*T2*^ activity is also detected in CDH5^+^ liver and spleen arteriole endothelium (Supplementary Fig. 2a and Supplementary Fig. 3a). *Cx40-creER*^*T2*^ activity is vascular-specific and not observed in either hematopoietic or stromal compartments in the liver or spleen (Supplementary Fig. 2c and Supplementary Fig. 3c). Moreover, direct comparison of *Cx40-creER*^*T2*^ and *Vegfr3-creER*^*T2*^ liver and spleen activity reveals anatomically-discrete vascular labeling, with *Cx40-creER*^*T2*^ marking typical large arteries (Supplementary Fig. 2a) in the liver and arteriole-enriched white pulp in the spleen (Supplementary Fig. 3a), while *Vegfr3-creER*^*T2*^ identifies characteristic sinusoidal vascular of the liver (Supplementary Fig. 2a) and sinus-enriched red pulp of the spleen (Supplementary Fig. 3a).

### *Vegfr3-creER*^*T2*^ and *Cx40-creER*^*T2*^ Activity Does Not Alter Hematopoietic Engraftment

To evaluate whether *creER*^*T2*^ induction in *Vegfr3-creER*^*T2*^ and *Cx40-creER*^*T2*^ mice have a deleterious effect on HSC-niche function, we performed a 1:1 competitive transplantation of WBM from tamoxifen-induced adult *Vegfr3-creER*^*T2*^ or *Cx40-creER*^*T2*^ mice (CD45.2^+^) with competitor WBM (CD45.1^+^) into lethally-irradiated CD45.1 recipients to examine HSC repopulation activity. Tamoxifen-induced C57BL/6 J wild type and pan-endothelial expressing *Cdh5-creER*^*T2*^ mice (CD45.2^+^) were used as controls for comparison. WBM cells derived from all three *creER*^*T2*^ lines displayed robust long-term multilineage hematopoietic reconstitution (Supplementary Fig. 6a, b), with no discernible off-target effects when compared to controls.

### Arteriole-specific MAPK-activation Drives HSC and Hematopoietic Dysfunction

**Fig. 3:**
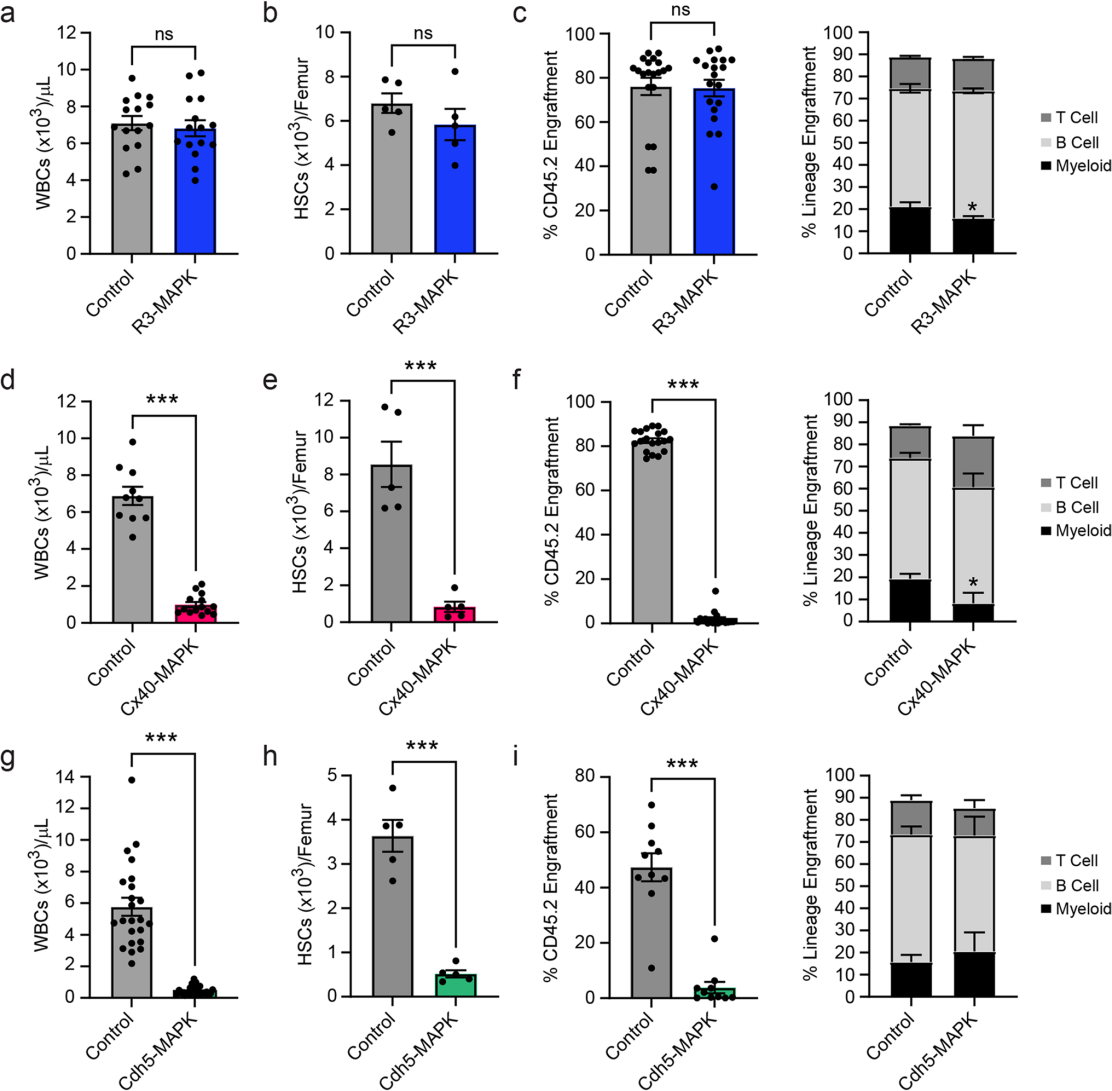
Hematopoietic defects observed in adult Cdh5-MAPK mice are driven by arteriole MAPK-activation. Adult *Mapk*^*fl/fl*^ mice crossed with sinusoid/transitional-specific *Vegfr3-creER*^*T2*^ (R3-MAPK; blue), arteriole-specific *Cx40-creER*^*T2*^ (Cx40-MAPK; red), or pan-endothelial *Cdh5-creER*^*T2*^ (Cdh5-MAPK; green) were induced with tamoxifen to activate MAPK signaling in BM endothelial subpopulations and assessed for hematopoietic dysfunction. Peripheral WBC counts in (**a**) R3-MAPK, (**d**) Cx40-MAPK, and (**g**) Cdh5-MAPK mice. Quantification of immunophenotypic HSCs in (**b**) R3-MAPK, (**e**) Cx40-MAPK, and (**h**) Cdh5-MAPK femurs. Total hematopoietic and lineage engraftment in competitive BM transplantation recipients from (**c**) R3-MAPK, (**f**) Cx40-MAPK, and (**i**) Cdh5-MAPK CD45.2^+^ donors. Tamoxifen-treated littermate *Mapk*^*fl/fl*^ mice serve as controls. Individual biological replicates are indicated per bar graph. Data is presented as AVE ± SEM. Significance is established using a Student’s t-test with *P* ≤ 0.05 (*), *P* ≤ 0.01 (**), and *P* ≤ 0.001 (***); ns = not significant

**Fig. 4:**
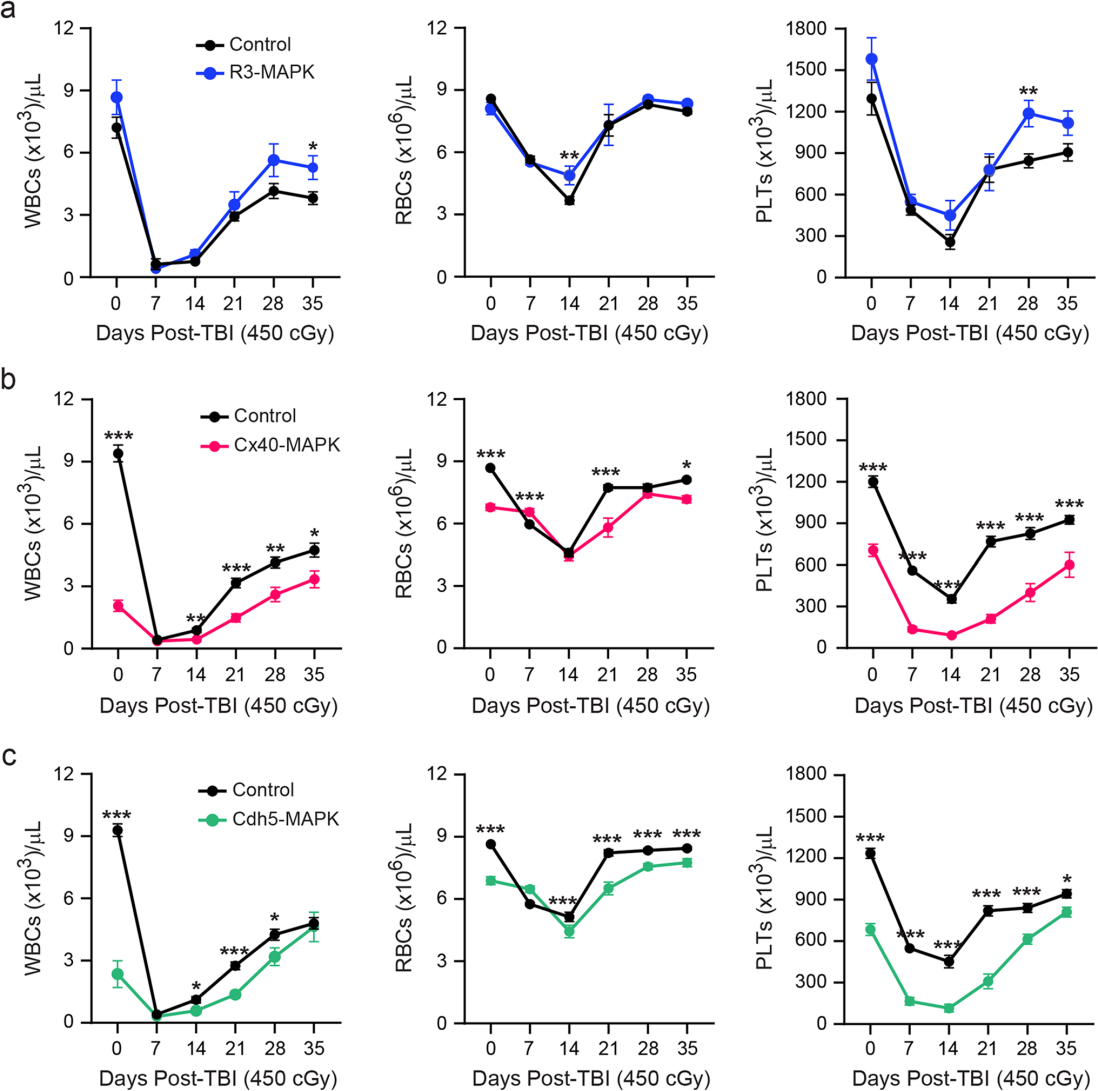
Hematopoietic recovery is inhibited in arteriole-specific Cx40-MAPK mice. Adult (**a**) sinusoid/transitional-specific R3-MAPK (blue), (**b**) arteriole-specific Cx40-MAPK (red), or (**c**) pan-endothelial Cdh5-MAPK (green) mice were subjected to myelosuppressive irradiation and assessed for peripheral hematopoietic recovery. Tamoxifen-treated littermate *Mapk*^*fl/fl*^ mice serve as controls. Cohort size for individual groups (N = Control mice/N = Experimental mice): R3-MAPK (14/10), Cx40-MAPK (18/9), Cdh5-MAPK (24/11). Data is presented as AVE ± SEM. Significance is established using a Student’s t-test at individual timepoints with *P* ≤ 0.05 (*), *P* ≤ 0.01 (**), and *P* ≤ 0.001 (***); ns = not significant

To validate the experimental utility of complementary BM sinusoidal/transitional and arteriole vascular-specific *creER*^*T2*^ mice, we generated *Vegfr3-creER*^*T2*^; *Mapk*^*fl/fl*^ (R3-MAPK) and *Cx40-creER*^*T2*^; *Mapk*^*fl/fl*^ (Cx40-MAPK) models in which *cre*-driven recombination of an upstream *loxP*-flanked stop cassette at the *Rosa26* locus induces constitutively-active *Map2k1* (S218D/S222D) signaling [20]. Chronic activation of the MAPK-ERK pathway in endothelium has been shown to drive vascular inflammation and HSC dysfunction in the BM microenvironment [15]. Adult *creER*^*T2*^; *Mapk*^*fl/fl*^ mice were induced with tamoxifen-supplemented feed and assessed for their respective contributions to endothelial-driven hematopoietic dysfunction in previously described *Cdh5-creER*^*T2*^; *Mapk*^*fl/fl*^ (Cdh5-MAPK) mice [15]. Arteriole-specific Cx40-MAPK mice developed a robust peripheral pancytopenia that phenocopied the previously observed dysfunction in Cdh5-MAPK mice, including a loss in white blood cells (WBCs), RBCs, and platelets (Fig. 3d, g and Supplementary Fig. 7b, c). Interestingly, sinusoidal/transitional-specific R3-MAPK mice showed no overt changes in peripheral blood counts (Fig. 3a and Supplementary Fig. 7a). Because pancytopenia is indicative of impaired HSC function in Cdh5-MAPK mice, we examined HSPC parameters in R3-MAPK and Cx40-MAPK models. Cx40-MAPK and Cdh5-MAPK mice displayed a significant decrease in both absolute numbers (Fig. 3e, h) and frequency (Supplementary Fig. 8d, g) of immunophenotypically-defined HSCs (lineage^−^cKIT^+^SCA1^+^CD48^−^CD150^+^) and HSPCs (lineage^−^cKIT^+^SCA1^+^) (Supplementary Fig. 8e, h), while R3-MAPK mice showed no changes (Fig. 3b and Supplementary Fig. 8a, b). Impaired HSC function in Cx40-MAPK mice was confirmed following competitive WBM transplantation; Cx40-MAPK mice displayed a significant decrease in multilineage engraftment (Fig. 3f), comparable to Cdh5-MAPK (Fig. 3i), while R3-MAPK engraftment was unaffected (Fig. 3c). Impaired HSPC activity in a semi-solid methyl cellulose assay was also observed in Cx40-MAPK, but not R3-MAPK mice (Supplementary Fig. 8c, f, i). Furthermore, Cx40-MAPK mice demonstrated a comparable delay in hematopoietic recovery to Cdh5-MAPK mice following a myelosuppressive dose of total body irradiation (Fig. 4b, c), while R3-MAPK mice were largely unaffected (Fig. 4a).

## Discussion

Vascular-directed *cre* mice are a vital tool to unravel the complex BM EC-instructive mechanisms that modulate HSPC function in vivo. However, model-specific differences in *cre* activity can have a profound impact on the interpretation of experimental phenotypes [10, 51]. In this study, we set out to characterize congenic C57BL/6 J *cre*-expressing mice models that (1) allow for inducible *cre*-mediated recombination in adult BM EC subsets, bypassing the potential pitfalls of embryonic HSC involvement, (2) avoid off-target *cre* activity in HSPC-supportive BM perivascular niche cells, and (3) define non-overlapping arteriole, transitional, and sinusoidal BM EC *cre* activity. In reporter mice, tamoxifen-treated *Vegfr3-creER*^*T2*^ and *Cx40-creER*^*T2*^ efficiently labeled discrete adult BM sinusoidal/transitional and arteriole EC populations, respectively, while avoiding hematopoietic and stromal involvement. *Vegfr3-creER*^*T2*^ and *Cx40-creER*^*T2*^ also specifically label discrete sinusoidal and arteriole endothelium in secondary hematopoietic tissues, including the liver and spleen, with no detectable activity in non-endothelial populations. Functionally, we demonstrated that *Vegfr3-creER*^*T2*^ and *Cx40-creER*^*T2*^ mice were able to phenotypically identify and segregate arteriole-specific activation of MAPK signaling as the source of hematopoietic dysfunction previously reported using a vascular-specific pan-endothelial *Cdh5-creER*^*T2*^ driver in adult animals [15].

While the identified *Vegfr3-creER*^*T2*^ BAC-transgene insertion point was mapped to an unplaced genomic segment and does not appear to directly disrupt any protein-coding genes, *Cx40-creER*^*T2*^ knock-in animals [26] disrupt the endogenous *Cx40* allele. However, the cardiovascular system in *Cx40*^+/-^ knockout mice display no reported gross differences when compared with wild type littermates [52–54]. Nonetheless, *Cx40-creER*^*T2*^ and *Vegfr3-creER*^*T2*^ mice should be maintained as heterozygotes to avoid potential complications due to excessive cre activity or loss of gene function at the transgene insertion loci. Because *cre* recombinase activity in murine model systems have been implicated in *loxP*-independent cellular cytotoxicity [55, 56], we also examined the potential effect of BM EC subset-specific creER^T2^ induction on HSC activity. Competitive transplantation of WBM from *Vegfr3-creER*^*T2*+^ or *Cx40-creER*^*T2*+^ tamoxifen-inducted animals demonstrated that creER^T2^ activity in these models does not impair hematopoietic reconstitution.

### Supplementary Information

Below is the link to the electronic supplementary material.

Supplementary file1 (TIF 11995 KB)

Supplementary file2 (TIF 19997 KB)

Supplementary file3 (TIF 19997 KB)

Supplementary file4 (TIF 13433 KB)

Supplementary file5 (TIF 11948 KB)

Supplementary file6 (TIF 9527 KB)

Supplementary file7 (TIF 14804 KB)

Supplementary file8 (TIF 14355 KB)

Supplementary file9 (XLSX 15 KB)

## Data Availability

All primary data generated in this study are available from the corresponding authors upon reasonable request.
