# Supplementary figures and images for: Complementary and Inducible creERT2 Mouse Models for Functional Evaluation of Endothelial Cell Subtypes in the Bone Marrow
Source: Stem Cell Rev Rep. 2024 Mar 4;20(4):1135–49. doi: 10.1007/s12015-024-10703-9 (PMC11087254; doi:10.1007/s12015-024-10703-9)

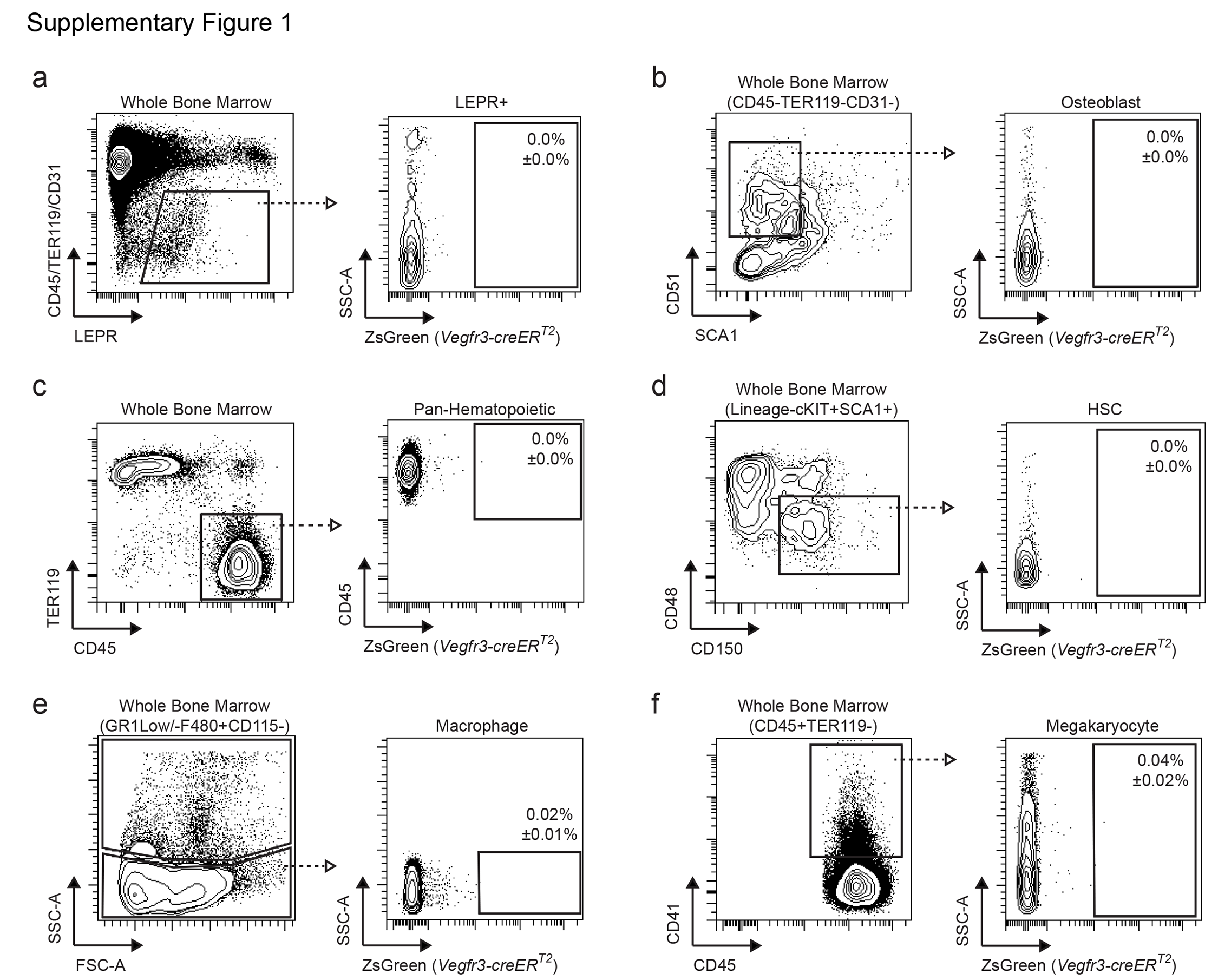

Supplement: Supplementary file 1 — Supplementary file1 (TIF 11995 KB) [file 12015_2024_10703_MOESM1_ESM.tif]

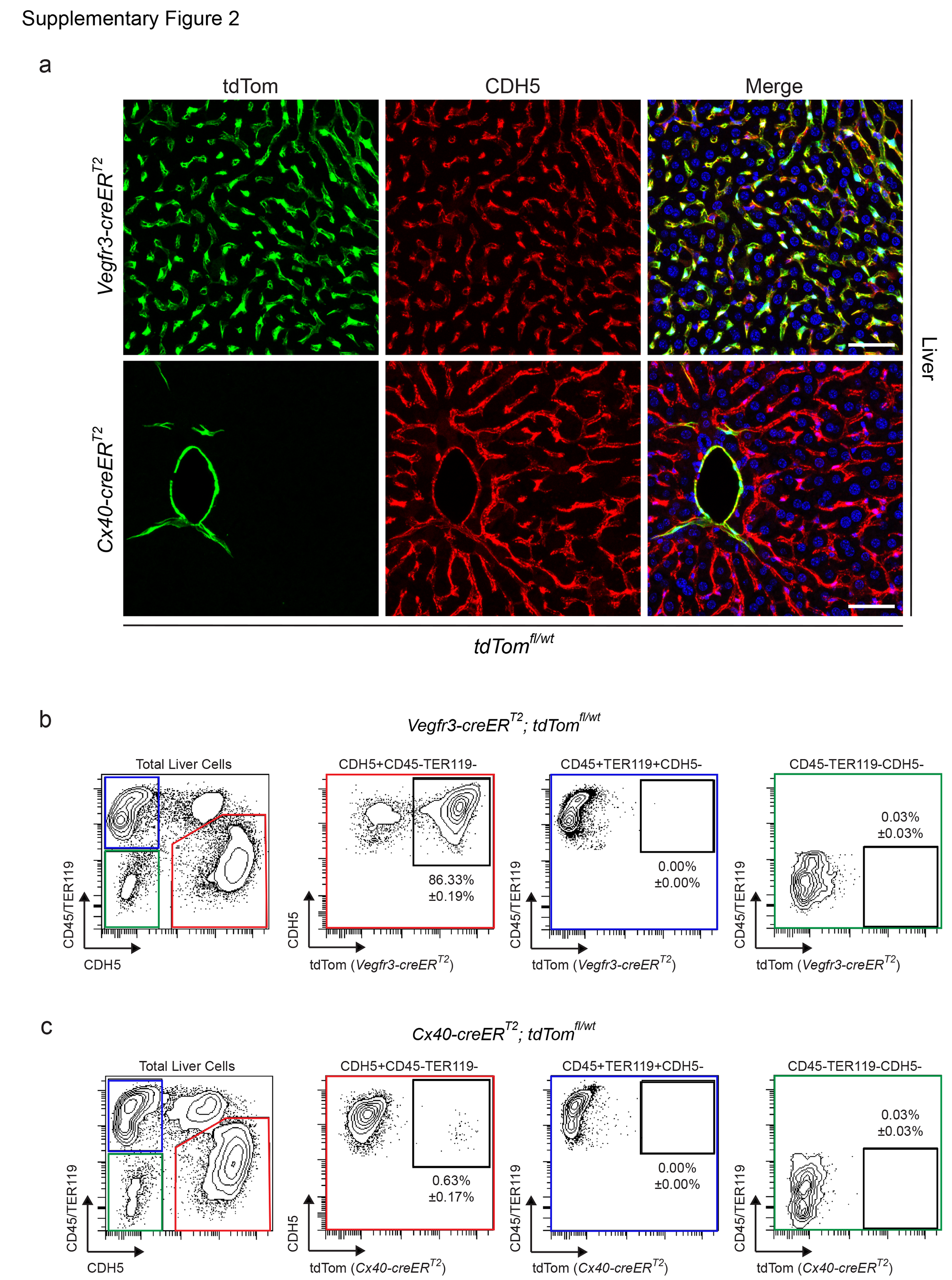

Supplement: Supplementary file 2 — Supplementary file2 (TIF 19997 KB) [file 12015_2024_10703_MOESM2_ESM.tif]

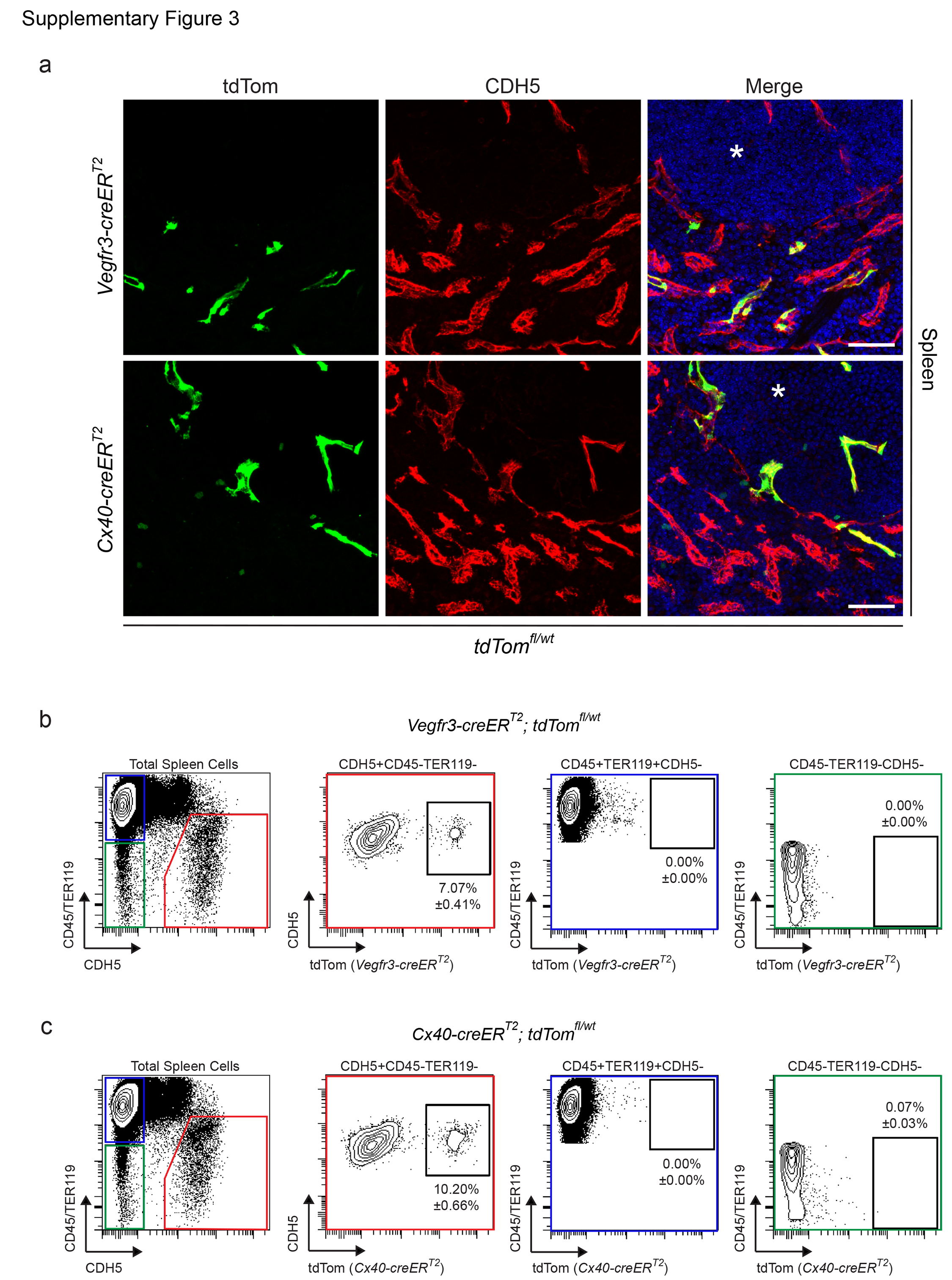

Supplement: Supplementary file 3 — Supplementary file3 (TIF 19997 KB) [file 12015_2024_10703_MOESM3_ESM.tif]

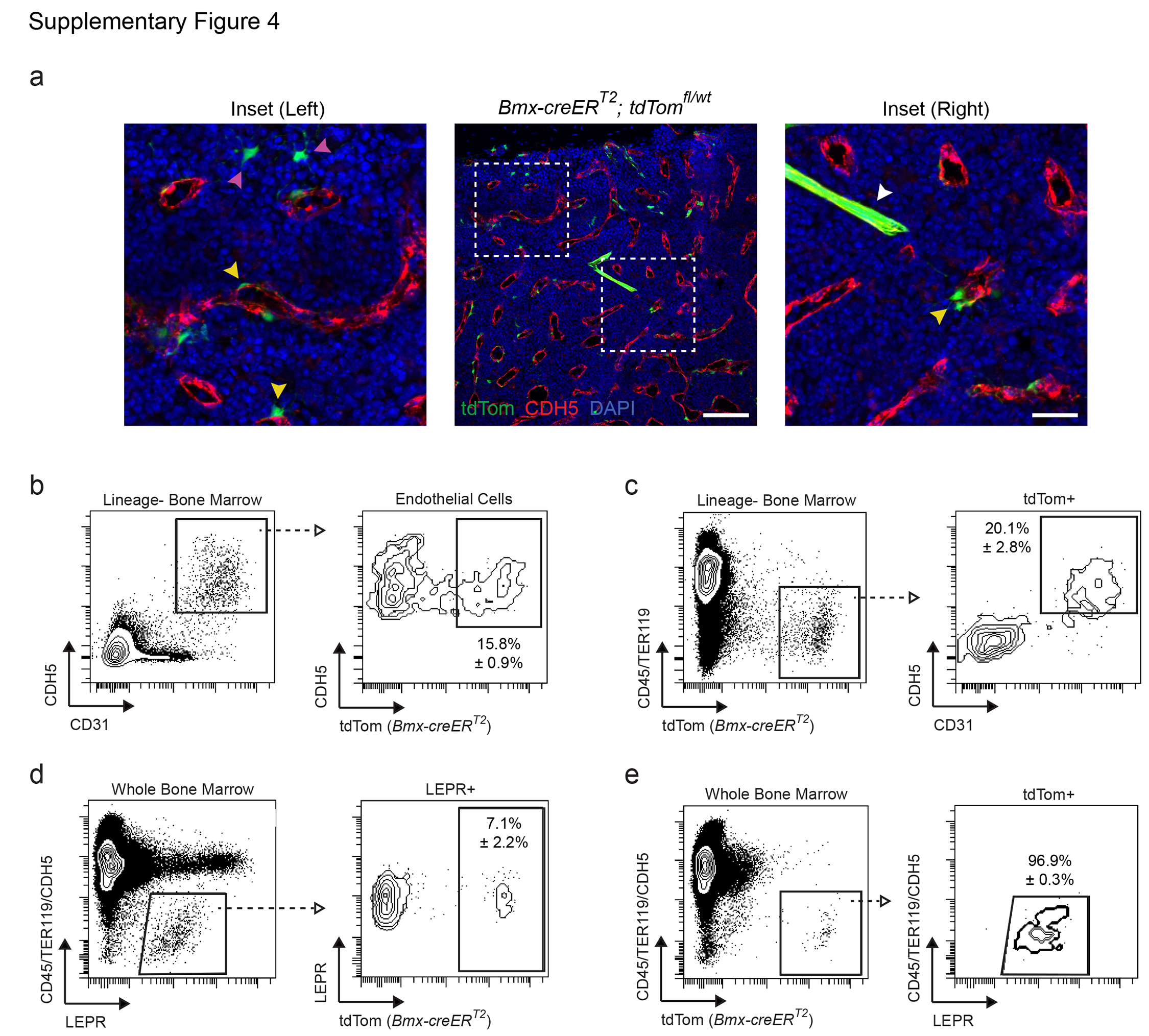

Supplement: Supplementary file 4 — Supplementary file4 (TIF 13433 KB) [file 12015_2024_10703_MOESM4_ESM.tif]

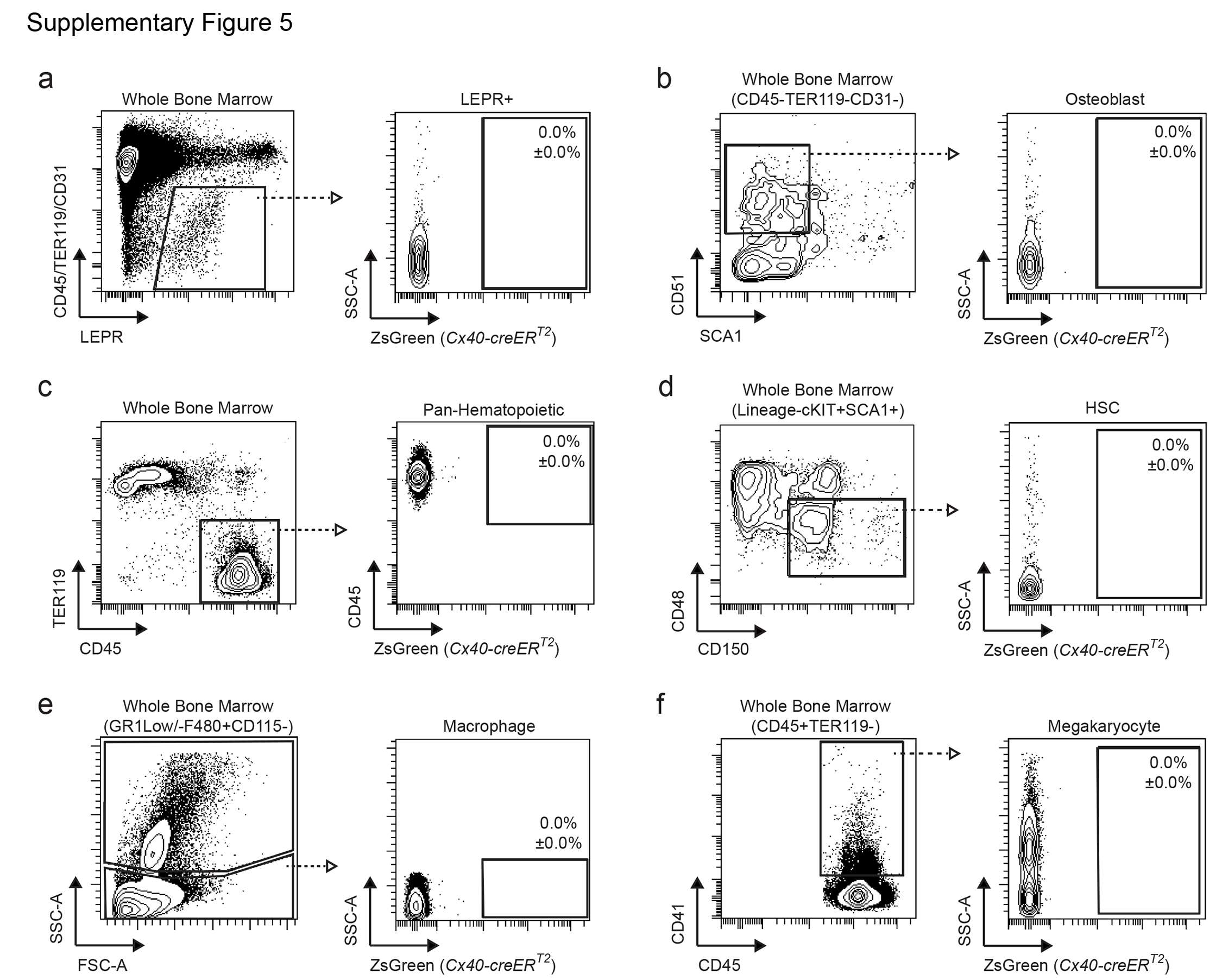

Supplement: Supplementary file 5 — Supplementary file5 (TIF 11948 KB) [file 12015_2024_10703_MOESM5_ESM.tif]

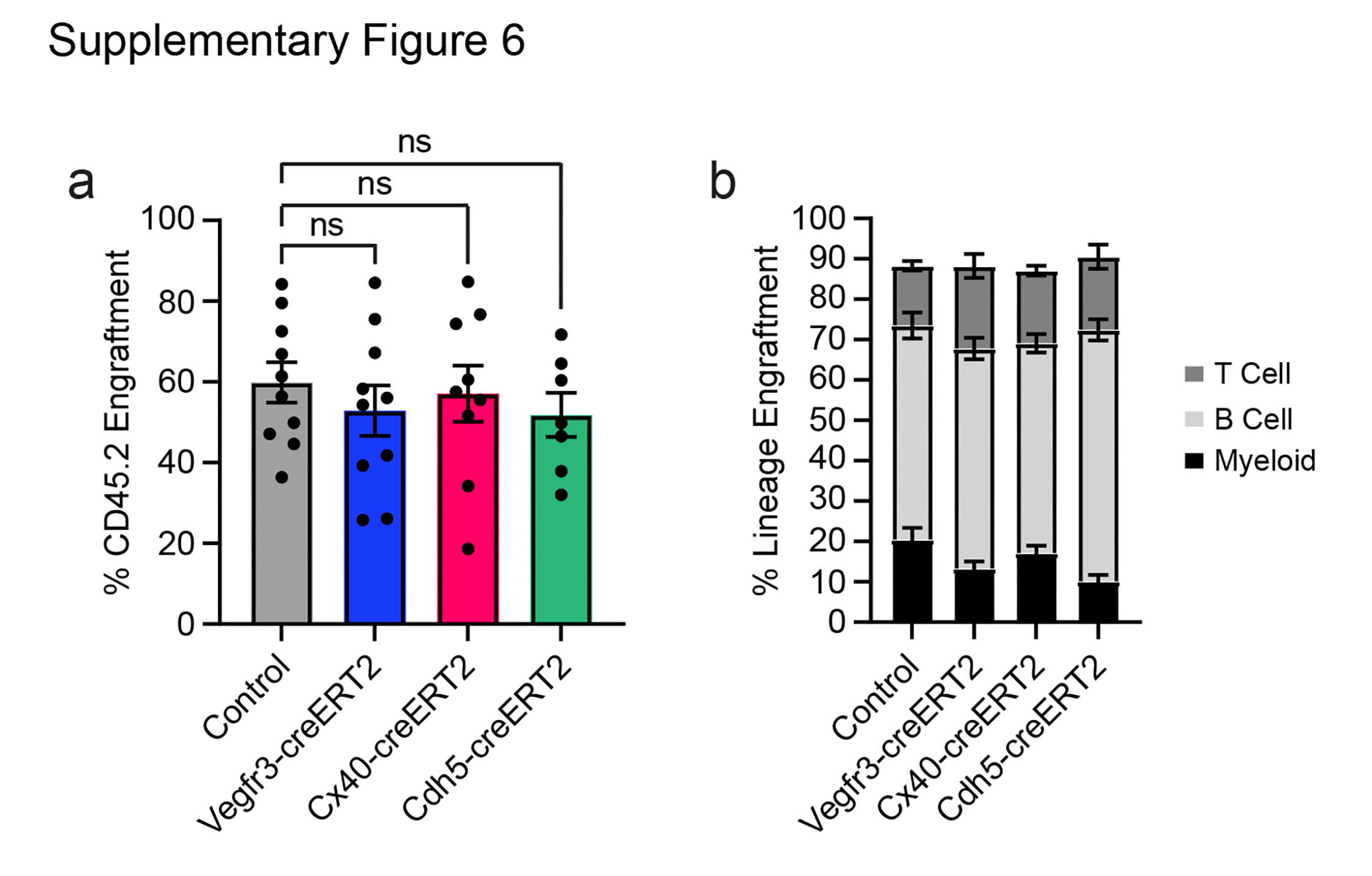

Supplement: Supplementary file 6 — Supplementary file6 (TIF 9527 KB) [file 12015_2024_10703_MOESM6_ESM.tif]

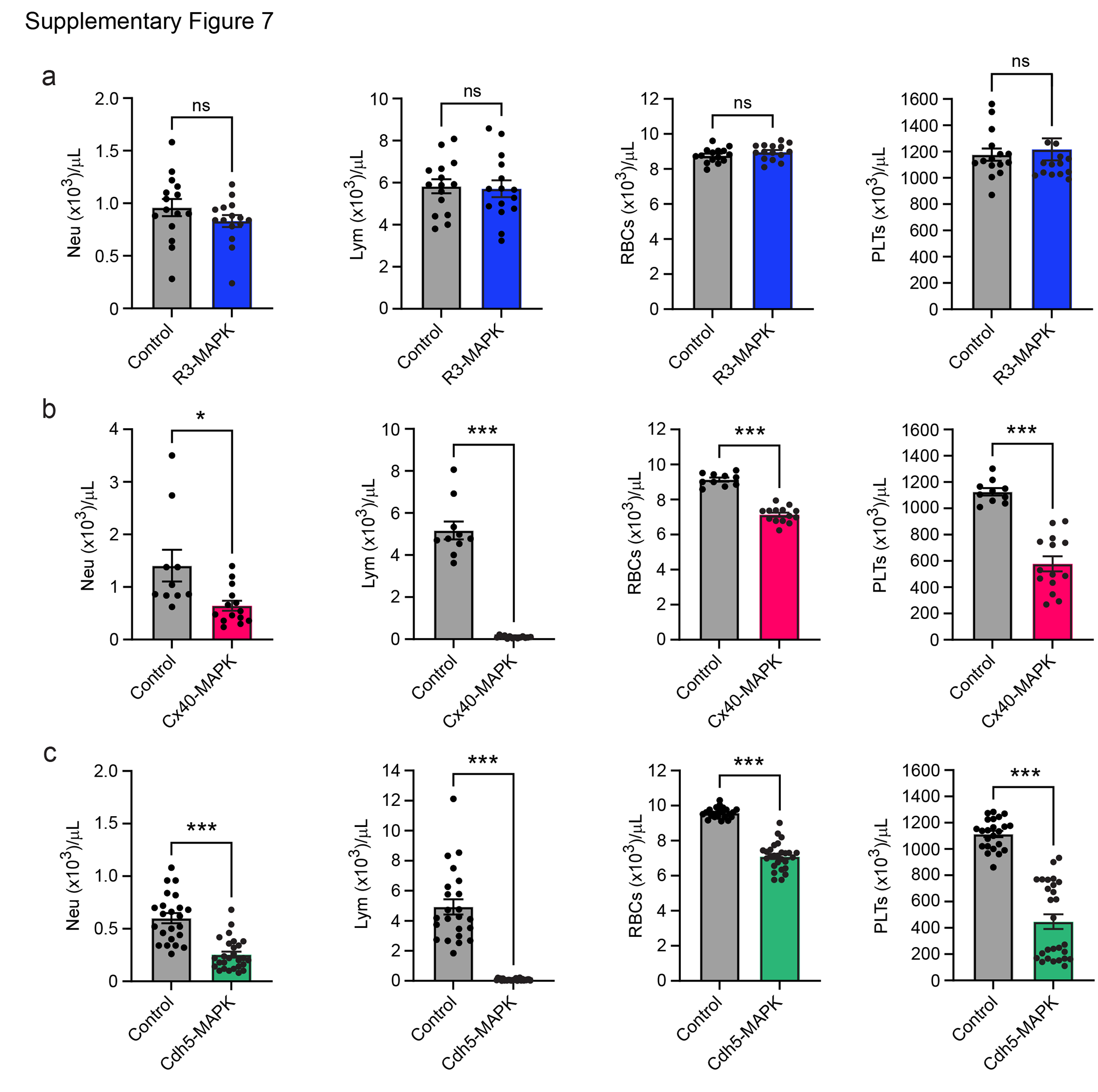

Supplement: Supplementary file 7 — Supplementary file7 (TIF 14804 KB) [file 12015_2024_10703_MOESM7_ESM.tif]

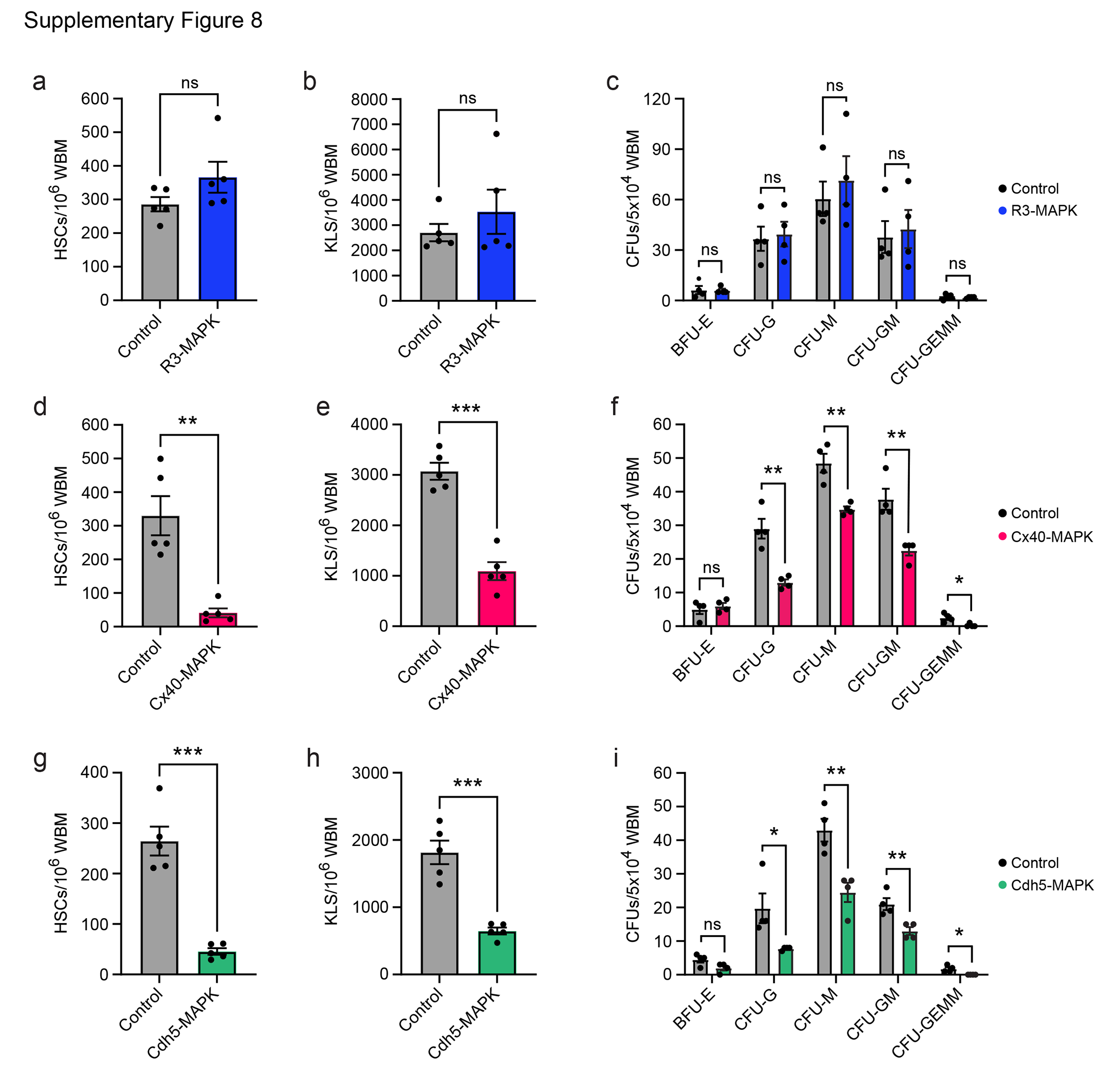

Supplement: Supplementary file 8 — Supplementary file8 (TIF 14355 KB) [file 12015_2024_10703_MOESM8_ESM.tif]
